# Implementing Brief Interventions in Health Care: Lessons Learned from the Swedish Risk Drinking Project

**DOI:** 10.3390/ijerph8093609

**Published:** 2011-09-07

**Authors:** Per Nilsen, Sven Wåhlin, Nick Heather

**Affiliations:** 1 Department of Medical and Health Sciences, Linköping University, Linköping 581 83, Sweden; 2 Swedish National Institute of Public Health, Östersund 831 40, Sweden; E-Mail: sven.wahlin@fhi.se; 3 School of Life Sciences, Northumbria University, Newcastle upon Tyne NE1 8ST, UK; E-Mail: nick.heather@northumbria.ac.uk

**Keywords:** alcohol, brief intervention, secondary prevention, public health, risk drinking, implementation, continuing professional education

## Abstract

The Risk Drinking Project was a national implementation endeavour in Sweden, carried out from 2004 to 2010, based on a government initiative to give alcohol issues a more prominent place in routine primary, child, maternity and occupational health care. The article describes and analyses the project. Critical factors that were important for the results are identified. The magnitude of the project contributed to its reach and impact in terms of providers’ awareness of the project goals and key messages. The timing of the project was appropriate. The increase in alcohol consumption in Sweden and diminished opportunities for primary prevention strategies since entry to the European Union in 1995 have led to increased expectations for health care providers to become more actively involved in alcohol prevention. This awareness provided favourable conditions for this project. A multifaceted approach was used in the project. Most educational courses were held in workshops and seminars to encourage learning-by-doing. Motivational interviewing was an integral aspect. The concept of risk drinking was promoted in all the activities. Subprojects were tailored to the specific conditions of each respective setting, building on the skills the providers already had to modify existing work practices. Nurses were afforded a key role in the project.

## 1. Introduction

In the past 30 years alcohol prevention research has shifted towards a public health strategy. It has been recognized that most alcohol-related harm at a population level is attributable to the large group of hazardous and harmful drinkers who are at physical, psychological, or social risk from alcohol intake or are already experiencing harm, rather than individuals with severe alcohol-related problems or alcohol dependence [1]. Before this change in emphasis, health care providers were charged with identifying alcohol-dependent persons and referring them for specialized treatment. Today, providers are increasingly expected to become involved in identifying and intervening with drinkers who are not seeking help for alcohol-related problems, but who may attend general health care settings for reasons related to their drinking.

The World Health Organization (WHO) has been instrumental in this shift in focus from treatment of dependent drinkers to prevention targeting individuals at risk of alcohol-related harm. A WHO Collaborative Project on the *Detection and Management of Alcohol-Related Problems in Primary Health Care* began in the early 1980s. The first phase of this project led to the development of the Alcohol Use Disorders Identification Test (AUDIT), the first screening instrument specifically aimed at detecting hazardous and harmful drinking [2]. The WHO initiative also facilitated the development of a brief intervention (BI) approach to provide early intervention for non-treatment-seeking, non-alcohol-dependent drinkers in general health care settings. In this population, the goal of BI is usually low-risk drinking because a demand for abstinence would be a deterrent to behaviour change [3]. In a review of policy-relevant strategies and interventions, “brief intervention with at-risk drinkers” was rated as being among the most effective approaches, with high breadth of research support and cross-national testing [1].

The effectiveness of the BI approach in reducing hazardous and harmful alcohol consumption has been established in trials undertaken since the 1980s, both as part of Phase II of the WHO Collaborative Project (1987–1992) and in other independent research [4,5]. Consistent with the growing evidence base, BI research since the 1990s has increasingly shifted from a focus on production of evidence to studies that examine ways of incorporating these interventions into clinical practice. Hence, Phase III of the WHO project (1993–1999) investigated the impact of various dissemination approaches on the uptake and utilization of screening and BI materials [6] and the concluding Phase IV (1999–2006) sought to develop and evaluate projects aimed at achieving more widespread BI implementation to reduce alcohol-related harm [7]. Two initiatives with similar aims have been launched in the wake of the WHO project: the Primary Health European Project on Alcohol (PHEPA) with funding from the European Union (EU) and the International Network on Brief Interventions for Alcohol Problems (INEBRIA), which involves many of the researchers who took part in the WHO Phase III and IV projects as well as new recruits from PHEPA [8] and other, similar projects.

The Risk Drinking Project was a national BI implementation endeavour in Sweden, carried out from 2004 to 2010. The project was a Swedish government initiative with the objective of involving primary, child, maternity and occupational health care in alcohol-preventive work and giving alcohol issues a more prominent place in routine care. The project was launched against a backdrop of increasing alcohol consumption in Sweden since the country’s entry into the EU in 1995. EU membership has led to a weakening of policies and various control instruments, including limitations on private imports of alcohol and increased alcohol taxation, thus restraining the Swedish government’s ability to pursue a restrictive primary prevention strategy based on measures of proven effectiveness such as controls on price and availability of alcohol [1]. This development has made it more important to pursue an alternative policy that is backed up by evidence of effectiveness—the strengthening of secondary prevention of alcohol-related harm in health care.

This article describes the content and analyses the results of the Risk Drinking Project. Critical factors that were important for the results are identified as lessons learned. These lessons are then discussed in relation to findings from other BI implementation projects and broader research on implementation, continuing professional education (CPE) for health care providers and organizational change.

## 2. Description of the Project

The Risk Drinking Project can be described in terms of Donabedian’s classic triad of structure, process, and outcome. Important structural aspects include a project’s goals, target groups, organization, resources and problem analysis. Process refers to activities undertaken to achieve the goals. Outcomes are the results of the project, typically measured against the goals [9].

### 2.1. Structure: Goals

The project was named the Risk Drinking Project to make it clear that it did not address more severe alcohol problems such as alcohol dependence. The project’s CPE efforts emphasized the significance of the concept of risk drinking (the Swedish term for hazardous and/or harmful drinking), the importance of early detection and knowledge about possible health consequences due to alcohol consumption. It was recommended that alcohol issues be brought up in a natural context, in connection with the health problem presented by the patient or with other lifestyle issues. The project promoted a patient-centred approach to addressing alcohol.

A vision for the project was formulated as follows: “The issue of alcohol should have an obvious place in daily health care and should be raised as often as is motivated by its significance as a cause of ill-health.” This vision was an expression of the basic idea that alcohol should become an obvious issue in health care that is addressed to the same extent as other issues of major significance to patients’ health. The project goals were that health care providers should: possess good knowledge of alcohol and risk drinking issues; have positive attitudes to bringing up the alcohol issue and discussing the patient’s drinking habits with them; be confident in their own ability (self-efficacy) to address alcohol with patients; and believe that they can influence patient’s drinking habits.

### 2.2. Structure: Targets

The Risk Drinking Project was national and aimed at achieving a high coverage of Swedish health care providers to create broad awareness of the project and its goals and key messages. This was deemed necessary to sustain the activities after the end of the project and institutionalize the changes that were promoted. The project targeted employees within primary health care (general practitioners, residents in family medicine, district nurses), child health care (nurses), maternity health care (midwives) and occupational health care (occupational physicians and occupational nurses). Child and maternity health care are formally part of primary health care but are also independent entities with goals of their own.

National subprojects were formed for each of these target groups, with a project manager appointed for each subproject. There was also a project manager for the motivational interviewing (MI) [10] training since this was an integral part of all the subprojects.

Further subprojects were added when it became obvious that the project was being well-received: student health services and health care for university employees, with activities directed at students and those who work at universities; an Internet website which provided an interactive and anonymous resource for those concerned about their own drinking or someone else’s drinking habits. Some other initiatives added towards the end of the project period included a subproject on tobacco use and another concerning family centres, which are open houses for parents, with social, psychosocial and nursing support.

### 2.3. Structure: Organization

The Risk Drinking Project was led by a director who worked closely with the project managers for the various subprojects. The project managers were approved by a council for supervision, formed by the professional organizations: the Swedish Association of General Practice, the Swedish Society of Nursing, the Swedish Organisation for District Nurses, the Swedish Association of Midwives and the Swedish Association of Occupational Health and Safety. The council also included representatives from the WHO Health Promoting Hospitals, Confederation of Swedish Enterprise and the Swedish Association of Local Authorities and Regions (SKL).

The project was built up in cooperation with all 21 county councils in Sweden. Swedish county councils are entitled to levy taxes for health care and are responsible for CPE for health care providers. The project was affiliated with existing networks for CPE and other professional development in the targeted settings.

The national project managers were the project drivers and were responsible for organizing the project activities and handling publicity. They also trained local project leaders in the county councils. The local project leaders organized local CPE activities and produced local materials.

### 2.4. Structure: Resources

The national project had government funding of approximately 100 million SEK (10 million euros), which covered salaries for the project director and managers as well as costs for conferences and other national activities, and the other project components cost approximately 150 million SEK (15 million euros), which was provided in equal amounts by the government (75 million SEK) and the county councils (75 million SEK). Thus, the total project cost approximately 250 million SEK (25 million euros) from 2004 to 2010 (175 million SEK governmental funding and 75 million SEK from the county councils).

### 2.5. Structure: Problem Analysis

The Risk Drinking Project was preceded by a professional initiative launched in 1996, when a network of interested general practitioners within the Swedish Association of General Practice was formed. The network instigated an analysis of obstacles and opportunities to implement secondary alcohol prevention in Swedish health care. They developed a pedagogic model, the Risk Drinking Workshop, in which general practitioners were given the opportunity to reflect on their approaches to alcohol and health and learn to use their existing knowledge to bring up sensitive issues with patients. Approximately 150 practitioners had participated in a Risk Drinking Workshop by 2000.

The Risk Drinking Project revisited the problem analysis conducted by the Swedish Association of General Practice, but also reviewed a number of Swedish articles and reports on obstacles to secondary alcohol prevention. The focus was on Swedish studies, but international research was also reviewed. Barriers identified included a perceived lack of knowledge, training and education on alcohol-related issues, uncertainty in one’s ability to discuss alcohol-related issues, doubt about the effects of alcohol counselling and insufficient documentation in the form of screening instruments and information materials about alcohol. However, a positive factor was the broad MI training effort carried out in the 2000s in primary health care, as part of the work on smoking. The reports from this effort showed that MI was perceived as attractive and highly relevant to daily work with lifestyle issues within health care.

### 2.6. Process

In order to strive towards the vision, the project used several CPE strategies to achieve objectives for activity, knowledge, attitudes and self-efficacy. The main activities were different types of educational courses (workshops, seminars and lectures), conferences, network meetings and distribution of information and materials. Knowledge was provided on alcohol and risk drinking issues, the practical use of the AUDIT and hands-on skills training in MI. The information materials included manuals, AUDIT forms, presentations and brochures. AUDIT was selected as many providers were already familiar with the instrument, having encountered it in various CPE efforts. AUDIT was also selected because of its focus on identifying non-dependent drinkers in contrast to tools such as CAGE (acronym for Cutting down, Annoyance, Guilt and “Eye-opener”) [11] developed to detect more severe alcohol problems.

The courses for primary, child and maternity health care providers were primarily conducted within the county councils. An important part of the project was a train-the-trainers scheme, by which training was provided for instructors (*i.e.*, health care providers) who, in turn, trained providers in primary, child and maternity health care. A total of 326 providers in primary, child and maternity health care were trained to be trainers. Of these, 155 providers were trained to conduct MI workshops for other providers.

Four national Risk Drinking Project conferences with 120–180 participants each were held during the project period. These were primarily directed at those who were responsible for the work within the county councils. Further, ten smaller seminars with 50 participants each were directed at the people who planned the activities within primary, child and maternity health care.

With regard to occupational health care, 22 courses were carried out to teach the Risk Drinking Model: screening with AUDIT and a biological marker (carbohydrate deficient transferrin (CDT)), followed by feedback and BI. In total, 530 people within occupational health care participated in the Risk Drinking Model training. Approximately 900 people participated in 40 half-day and whole-day information seminars and seven network meetings for knowledge and experience exchange within occupational health care. Fourteen national conferences were held across the country in cooperation with the Confederation of Swedish Enterprise, the Swedish Association of Local Authorities and Regions, the employers’ association, Alecta, and the insurance firm, AFA. In total, 930 people participated in these conferences. In addition, 160 local and regional workplace seminars were held in cooperation with occupational health care units and workplaces nationwide, with approximately 6000 participants.

### 2.7. Outcomes

Success in reaching the project’s objectives for activity, knowledge, attitudes and self-efficacy was assessed in two cross-sectional surveys, a baseline questionnaire (in 2005–2006) and a follow-up questionnaire (in 2008–2009) completed by the targeted professional groups in primary, child, maternity and occupational health care. Overall, evaluations suggested that improvements occurred in activity, knowledge and self-efficacy; attitudes towards identifying risk drinking patients and counselling on alcohol issues were overwhelmingly positive from the outset and did not change much.

Evaluations suggested a consistent connection between the degree of alcohol prevention activity undertaken by the health care provider and how much CPE they had received in the handling of risky drinking. Those with more training were more active. However, the study’s cross-sectional design does not allow simple conclusions concerning causal relationships to be made. It may be the case that those who were already most active were also those who participated most in the CPE.

Within primary health care, knowledge, self-efficacy and activity improved among general practitioners, interns and district nurses. However, it was the district nurses’ knowledge, ability and activity that consistently improved most.

A similar pattern was discernible in occupational health care. Improvements were generally somewhat smaller than in primary health care but occupational health care had already shown a higher level of knowledge, self-efficacy and activity at the baseline measurement. Occupational physicians and, especially, occupational nurses were very active in discussing drinking habits with their patients. Such issues are included in the lifestyle and health examinations that are common in this setting. Overall, it was nurses who increased their knowledge regarding counselling risk-drinking patients and improved their self-efficacy concerning the handling of risk drinking.

Alcohol prevention efforts in child and maternity health care also improved in various respects. Child health care nurses became more knowledgeable on giving advice to parents with at-risk consumption. Midwives in maternity health care also increased their knowledge, mainly becoming better at identifying patients with at-risk consumption.

Are there alternative explanations for these favourable results? There are many potential factors that may have influenced the results because projects of this kind are implemented in a social context rather than under experimental conditions with good control of independent variables. However, there were no other concurrent alcohol-related CPE projects. And while social norms and values change over time in a society, it seems unlikely that any major changes occurred in the relatively short period during which the Risk Drinking Project was conducted.

The drop-out rate should be taken into consideration when interpreting the results. The response rate varied across the various personnel categories, from an impressive 80% for occupational health care nurses (follow-up questionnaire) to a more modest 43% for child health care nurses (baseline questionnaire). Response frequencies for most of the categories were higher in the follow-up than in the baseline questionnaire, which implies an increased interest in participating in the project evaluation and/or a bias towards more responses from those most interested in the project. Individuals’ baseline and follow-up responses could not be linked to analyze changes at the individual level because the questionnaire was anonymous. The drop outs were not analyzed to assess ways in which those who did not respond might have differed from those who responded to the questionnaire. Despite these weaknesses, a total of nearly 10,000 providers responded to the baseline questionnaire and as many to the follow-up questionnaire. Hence, the results can be considered relevant to a major part of Swedish health care.

To what extent are these findings corroborated by other research undertaken in Sweden? Studies [12] into the extent to which patients receive lifestyle advice from physicians and nurses in Swedish health care have observed minimal changes over time, but it is not known whether alcohol has become more frequently addressed or not. Increased alcohol-preventive activity in health care can be expected to yield increased detection of alcohol dependence and other problems attributable to alcohol. An unpublished study [13] found that the number of alcohol-related diagnoses recorded in medical journals in western Sweden (population approximately 1.5 million) increased by 9% between 2006 and 2008. More research is needed to determine long-term changes in alcohol-preventive activity in health care and the long-term effects on hazardous and harmful drinking in Sweden.

## 3. Lessons Learned

What lessons can be learned from the generally favourable outcomes of the Risk Drinking Project? Some key factors were anticipated beforehand and constituted important elements of the project structure and process, although their importance could not be predicted with certainty. Other factors are more appropriately described as insights gained during the course of the project or conclusions derived from project evaluations. The nine lessons described here are not mutually exclusive but are interdependent and overlapping; these factors have, to varying degrees, contributed to the positive outcomes of the project.

The nine lessons draw on findings regarding various subprojects presented in six scientific papers [14–19], one report by the Swedish National Institute of Public Health concerning the MI training component of the project [20], and two overview reports by the Swedish National Institute of Public Health [21,22]. Observations are also based on the authors’ informal knowledge and overall familiarity with the project.

### 3.1. Project Magnitude

The Risk Drinking Project had substantial governmental funding, lasted 6 years and applied a broad settings approach, targeting primary, child, maternity and occupational health care. Further subprojects were added along the way. Unquestionably, the sheer magnitude of the project contributed to its reach and impact in terms of health care providers’ awareness of the project and its goals and key messages. The broad project exposure made it possible to firmly establish risk drinking and alcohol prevention as important issues for a large proportion of the providers in major sections of the Swedish health care system.

### 3.2. Sense of Ownership

The Risk Drinking Project was designed to take account of informal criticisms that had been directed at previous CPE efforts concerning alcohol prevention in primary health care. These efforts were often seen as being top down, failing to account for the voices of the “shop floor workers”. Negative descriptions such as “commando training” were commonly applied to those efforts. In the Risk Drinking Project, a sense of ownership emerged from the active involvement of national and local project managers, as significant numbers of providers in the targeted health care settings felt committed to the project (perhaps most notably in child and maternity health care, where participation in the courses was almost 100%). A sense of ownership refers to the belief of a group of people that an issue or a project belongs to them. How intensively and extensively the people are involved in defining the project, the planning process and the implementation will affect the sense of ownership [23].

The project leaders were instrumental in all phases of the project, *i.e.*, development, implementation and supervision. They were well connected with the professional organizations they represented and experienced full support from their respective organizations, giving them a strong mandate to act. The local project leaders in the county councils were also important because they possessed the local know-how and networks, making it possible to tailor initiatives to local conditions.

### 3.3. Contextual Adaptation

The Risk Drinking Project did not seek to implement a one-size-fits-all BI solution in the various settings. Instead, the different subprojects were tailored to the specific conditions of each respective setting. An important ambition was to build on the skills the providers already had and to develop and modify existing work practices rather than implement new, different routines. It was deemed important that the proposed routines and practices were contextually adapted to the everyday reality of the health care providers and to the settings in question.

Physicians and nurses in primary and child health care were trained in bringing up the alcohol issue in a patient-centred context and in ways that suited various clinical situations and their own practice and experience. No specific, predetermined model of BI was promoted. Universal screening was not advocated for use in these settings.

In contrast, the occupational health care providers were trained to use AUDIT and a biological marker as screening instruments. This was done in the context of other health and lifestyle questionnaires that are commonly used in occupational health care (unlike primary health care).

Midwives in maternity health care also adopted the use of AUDIT, but more as a pedagogic tool than as a screening instrument. Rather than asking about the pregnant woman’s drinking *during* the pregnancy, the project introduced a modified routine whereby she fills in the AUDIT questionnaire concerning her alcohol use in the year *preceding* the pregnancy, which has been shown in research to be an important predictor for drinking during pregnancy [19]. The AUDIT results then provide the basis for a discussion. The new routine accounted for the fact that it can be difficult to obtain reliable self-reports of alcohol use during pregnancy by means of direct questions about a pregnant woman’s current drinking. The new routine had strong support from the midwives and was quickly adopted by nearly every midwife who participated in the training and education. The project also instigated a new nationally implemented routine whereby midwives bring up alcohol issues at an early first meeting with the pregnant woman.

### 3.4. Modifying the Culture

The Risk Drinking Project actively promoted the concept of risk drinking in all the CPE activities that were undertaken. The aim of the risk drinking message was to reframe alcohol issues for a broadened understanding of the full spectrum of alcohol problems and increased recognition that the key issue in secondary alcohol prevention is detecting and intervening with non-dependent hazardous and harmful drinkers. The project appears to have succeeded in modifying the prevailing alcohol problem culture, which traditionally has placed more emphasis on severe alcohol problems, by making risk drinking a more widely recognized and better understood concept. The culture of an organization or a group of people is the shared beliefs, attitudes, norms and values of the people in the organization or group; it is “how we do things round here” [24].

### 3.5. Involvement of Nurses

Nurses were afforded a key role in the Risk Drinking Project as they were highly involved in the CPE in all settings. They responded enthusiastically to the project. Evaluations showed that the improvements in their knowledge, self-efficacy and alcohol-preventive activity were pronounced in primary and occupational health care. For example, the proportion of occupational health care nurses who rated themselves as “very knowledgeable” concerning counselling on alcohol issues and “very efficient” in achieving changes in patients’ alcohol consumption more than doubled. Physicians in these settings also achieved improvements but they generally started from higher levels of perceived knowledge and self-efficacy, which meant that their change was less dramatic than that for the nurses. The overall results suggested that nurses were highly motivated to take on a more active role in alcohol prevention.

### 3.6. Multifaceted CPE Approach

The Risk Drinking Project used a multifaceted approach to target many of the obstacles to secondary alcohol prevention that were identified in the problem analysis. The CPE activities involved various forms of educational courses (workshops, seminars and lectures), national and regional conferences, different types of network meetings and distribution of printed information and materials. The project’s multifaceted CPE approach contributed to reducing many of the obstacles to providing BI, including insufficient knowledge, training and education in alcohol-related issues, uncertainty in one’s ability to discuss alcohol-related issues, doubt about the effects of alcohol counselling, and poor documentation in the form of information materials about alcohol.

### 3.7. Active CPE

Many of the educational courses in the Risk Drinking Project can be characterized as active and engaging, with many being held in workshops and seminars to encourage learning-by-doing. Reflection based on the health care providers’ own experiences of addressing alcohol issues with their patients was also an integral part of training and education. Traditional didactic approaches were used more sparingly, primarily to convey more fact-based knowledge, e.g., concerning the likely influence of alcohol on some of the more common diagnoses in primary health care.

### 3.8. Learning MI

Many health care providers in the Risk Drinking Project learned to use MI during the scope of the project. MI training was used in all settings (and all subprojects). An evaluation showed that those who had taken part most in MI training were most active in bringing up the alcohol issue with patients. While again causal inference is not possible, these findings nevertheless suggest that more MI training was associated with more alcohol-preventive activity. Informal reports from project managers and health care providers also suggested that MI was widely regarded as an important tool for opening and managing conversations about alcohol.

### 3.9. Timing of the Project

The timing of the Risk Drinking Project appears to have been very appropriate, thus indirectly contributing to the positive outcome. The combination of increased alcohol consumption in Sweden and diminished opportunities for primary prevention strategies since EU entry in 1995 has led to increased expectations for health care providers to become more actively involved in alcohol prevention. Swedish health care providers have increasingly recognized that their role entails identifying and intervening with patients who are not seeking help for alcohol-related problems but who may attend health care without much or perhaps any awareness that their drinking habits are a potential problem. The providers’ recognition of the relevance of this issue provided favourable conditions for the learning in the project.

The project was also well timed in relation to growing awareness of the public health importance of lifestyle issues, which has occurred over the last few decades. Health-compromising behaviours and risks such as alcohol consumption, sedentary lifestyle, poor dietary habits, overweight and tobacco use have been more widely acknowledged as important causes of mortality and morbidity, imposing a significant burden on the health care system in Sweden and elsewhere. The need for a stronger preventive and health-promoting health care focus has received greater attention, preparing the ground for a project such as the Risk Drinking Project.

Changes in social norms and values concerning alcohol use in the Swedish population have taken place in conjunction with increasing globalization and Swedish EU membership. Fewer Swedish health care providers today than before believe that alcohol is a sensitive issue they want to avoid addressing with their patients or are afraid of provoking negative reactions from patients if they bring up the alcohol issue. While therapeutic relationship factors still influence alcohol-preventive activity, there is no question that many of the previously reported obstacles to addressing alcohol or providing alcohol interventions in general health care are less relevant today than just 10–15 years ago.

## 4. Discussion

To what extent do the lessons learned from the Risk Drinking Project correspond with experiences from other BI implementation projects described in the scientific literature? Such projects have been carried out in Spain [25,26], England [26,27], South Africa [28], Brazil [28], New Zealand [26], the USA [29] and in seven other countries that were part of Phase IV of the WHO Collaborative Project (in addition to the aforementioned projects in Spain and England): Denmark [30], Finland [31], France [32], Italy [33] and Russia [34]. The lessons of the Risk Drinking Project can also be interpreted in light of findings from broader CPE, implementation and organizational development research.

The Risk Drinking Project was a national project based on a government initiative, in contrast to most other BI implementation projects that have been described in the literature. Few other projects appear to have had the same degree of support from authorities at local, regional or national levels. A notable exception is the New Zealand project, Tobacco, Alcohol and other Drugs (TADS), a comprehensive national initiative launched in 1995 with a focus on training primary health care providers in the use of AUDIT and MI principles for providing alcohol interventions. TADS later diversified into a broad generic lifestyle educational project [26]. Limited governmental interest at various levels and poor coordination with health agencies’ campaigns were reported in many of the projects in Phase IV of the WHO Collaborative Project [7]. This lack of engagement was attributed to the long-standing failure by governments to recognize the full extent of alcohol-related harm. However, there have been positive signs in several countries that governments are beginning to take the aim of implementing BI in the health care system more seriously [8].

The Risk Drinking Project had major funding, again unlike many BI implementation projects described elsewhere. Indeed, a recurrent problem in Phase IV of the WHO Collaborative Project was the difficulty of obtaining sufficient funding from national bodies. Consequently, only seven of the 12 countries in the project were able to implement and evaluate BI implementation projects [7]. Although the Risk Drinking Project relied on central governmental funding, the regional county councils had to provide an equal amount of money to obtain the money from the government. This arrangement was a way to ensure local and regional commitment to the project. Funding itself does not solve the BI implementation problem, but financial resources can provide the opportunity to mount projects with long-term viability, which is often a prerequisite for achieving lasting changes in knowledge, attitudes and behaviour [35]. Moreover, project participants are more likely to commit themselves to initiatives that are not seen as just another short-lived temporary experiment [36].

In all its aspects, the Risk Drinking Project promoted awareness of the risk drinking concept and stressed the importance of early detection of risk drinkers and of avoiding paternalistic approaches to addressing alcohol. This focus made it possible to reframe the alcohol problem and foster a modified culture concerning alcohol problems in large segments of the Swedish health care system. This process of cultural change had started well before the project was launched, but it is likely that the project accelerated its pace. Communication of a clear vision is generally considered an important factor in achieving successful organizational development and change work [37].

The Risk Drinking Project involved health care providers to a greater degree than most other BI implementation projects described in the literature. The engagement and prominent roles of many practitioners seemed to engender a sense of project ownership and continuing responsibility for the project by large numbers of providers. This contributed to the project being perceived as having a bottom-up orientation rather being viewed as a top-down *diktat* despite the fact that it originated from a government decision. Some BI implementation projects in the literature have been more researcher led, with limited input by the practitioners in various project stages. The danger of conducting overly efficacy-oriented BI implementation research has been noted [5]. BI implementation research and practice do not benefit from projects that depend on researchers for their sustainability or are too complex or costly for continued use in practice, if found effective. The Risk Drinking Project points to the relevance of researchers collaborating with practitioners and policymakers to pursue more practice-based translational research that combines bottom-up engagement to ensure provider input and top-down decisions and policy, to provide legitimacy for the alcohol-preventive work.

The alcohol-preventive practices promoted in the Risk Drinking Project were adapted to the health care providers’ existing routines and were sensitive to the conditions of the various settings. A similar approach was used in the New Zealand TADS project, which favoured alcohol interventions tailored to the specific health care setting culture. TADS promoted opportunistic interventions that were often a series of conversations or brief comments that occurred over a series of meetings that the patient attended for other reasons [26]. The changes sought in the Risk Drinking Project were incremental, *i.e.*, the proposed new way of working did not require the practitioners to move too far from what they were used to or felt comfortable with. Research on organizational development has consistently shown that implementation of relatively small changes is far more likely to succeed and be sustained than attempts to introduce more radical changes [37].

The primary health care subproject of the Risk Drinking Project promoted the idea that alcohol issues should be brought up in connection with an appropriate medical condition likely to be associated with or caused by the patient’s alcohol habits. No specific BI structure or theory was promoted. Instead, health care providers were encouraged to enquire about and discuss patients’ alcohol habits in a manner that suited their own practice and experience. A very similar approach was taken in the TADS project in New Zealand, which assumed that physician-led interventions should be triggered when an appropriate medical condition arose that was likely to have been caused by at-risk drinking [26].

There has been a debate among BI implementation researchers about the use of screening as a prelude to BI. Early BI implementation research was based on the assumption that all patients attending primary health care facilities should be screened and a physician (or someone from another professional category) should offer interventions to all patients screening positive for hazardous or harmful drinking [2]. This type of screening procedure has been the norm in BI research that has investigated the efficacy and effectiveness of BI in reducing patients’ alcohol consumption [4]. However, providers and researchers have increasingly questioned this blanket screening approach, which many consider unrealistic on workload grounds and potentially harmful for the therapeutic provider-patient relationship [38]. This development underscores the importance of conducting more BI research under realistic circumstances. Closing the widely acknowledged gap between production of research findings and their application in routine practice requires a better understanding of real-world health care practice.

Projects such as the Risk Drinking Project and TADS suggest that there will be considerable variation in how alcohol-preventive work is carried out. The danger with this practice-based approach is, of course, that interventions implemented in practice might deviate substantially from those that have been shown to be effective in research, to the point where evidence-based practice becomes a misnomer. A recent population-based study [39] found that nearly two-thirds of the alcohol conversations in Swedish health care lasted less than 1 minute and only 6% of the interventions were longer than 5 minutes. Similar results concerning the duration of primary health care alcohol conversations have been observed in Finland [40]. In comparison, the Cochrane review by Kaner *et al*. [4] included very few studies that investigated BIs shorter than 10 minutes (not including screening) and the mean duration of a BI in this review was in excess of 20 minutes. There is a need to strike a balance between the forms of intervention that have been validated as effective in carefully controlled research and making the implementation of these interventions consistent with the realities of existing practice.

The CPE activities undertaken in the Risk Drinking Project reached a large section of the Swedish health care system. Although similar projects have been carried out in many countries, few have been on as large a scale as the Swedish project. Despite differences in scope and contents, CPE projects have generally reported positive findings that demonstrate that trained providers are more active in providing alcohol interventions than non-trained providers [7,26,41]. There is empirical support for the sort of mass education provided in the Risk Drinking Project, as it has been shown that educational efforts are more likely to have an impact on professional practice if they involve many people at a workplace than if single individuals participate in courses and acquire knowledge and abilities that their colleagues and managers may not be particularly interested in [42,43]. The importance of involving more than just a few individuals in the workplace can also be explained with reference to contextual and informal learning perspectives, which have a collective and social view of learning. This means that educational efforts have greater impact if many people in a workplace participate in them since a major part of learning takes place informally in the everyday discussions with colleagues at work [44].

The Risk Drinking Project applied a multifaceted approach to target various barriers to BI implementation. Research on implementation interventions (which target health care providers) has generally shown that there are no magic bullets for achieving practice change. A range of implementation interventions can lead to behaviour change, but no single intervention is always effective for changing practice behaviour. Multifaceted approaches tend to be more effective than single interventions because they address multiple barriers to implementation [45] and may take advantage of a synergy that is assumed to exist between different components [46].

The Risk Drinking Project relied mostly on active and engaging educational efforts, many of which provided very hands-on conversational skills and techniques for use in patient encounters. Research on CPE for health care providers has shown that more demanding and active training and education is most effective in influencing the practice of providers. More passive approaches, such as sending information materials or holding lectures, are generally ineffective and are unlikely to result in behaviour change when used alone [47]. Many of the projects that were part of Phase III of the WHO Collaborative Project used passive strategies, including sending screening materials, and produced fairly small increases in alcohol-preventive activity [6,48]. Although active approaches are more likely to be effective than passive approaches, they are also more costly [49].

MI training was an essential part of the Risk Drinking Project. There was already great interest in MI before the project, as it has rapidly achieved widespread implementation in Sweden. MI has been actively supported by several state agencies and advocated in various governmental initiatives aside from the Risk Drinking Project. Although there is no distinctly better evidence for the effectiveness of MI than other approaches for influencing patients’ lifestyle behaviours, an important appeal of MI may be its wide application across many behavioural domains and client populations. MI is also compatible with many different preventive and treatment approaches, and this facilitates its integration into routine practice [50]. It has also been noted that many practitioners consider MI intuitively appealing because they view the MI principles to be consistent with how they want to work [51]. MI originated in the alcohol field [52] and may have particular relevance to alcohol prevention, as it has been shown to facilitate discussions concerning issues that can to some extent be perceived as sensitive or difficult to discuss [53]. The extent to which MI training has been used in other BI implementation projects is not clear because the contents of CPE efforts are not always explicitly described.

Nurses’ involvement was another important factor in the success of the Risk Drinking Project. Most BI implementation research hitherto has concerned physicians. However, studies in the United Kingdom and Sweden [54–56] have indeed suggested that nurses are an underutilized resource in alcohol-preventive work. They tend to be more favourably disposed to preventive work in general and have a more holistic perspective on patients than physicians, with more time to spend with patients. Interestingly, the New Zealand TADS project involved no less than 20 different professional groups exposed to potential hazardous and harmful drinkers, including dieticians, pharmaceutics and mental health workers [26]. There is a need for broader BI implementation research to investigate alcoholpreventive work that involves more than physicians and nurses.

The timing of the Risk Drinking Project played an important part in the favourable results. The project had elements of both proactive changes in response to perceived expectations for increased alcoholpreventive activity and reactive changes in response to changes in the environment that have already occurred. The timing of implementing change work and organizational changes is generally considered an important factor to achieve desired changes, although there are no simple generalizations as to *when* projects are best implemented [42]. As recognition grows of the full extent of the harmful effects of excessive alcohol consumption on society, it can be expected that there will be a matching increase in the need for secondary alcohol prevention in the health care system and a greater acceptance by government, practitioners and the general public of their role in combating alcohol-related harm.

Aside from the factors addressed here as lessons learned, it is also likely that the Swedish context of the Risk Drinking Project contributed to its favourable results. Sweden has 9 million inhabitants, with 83% living in urban areas and 94% belonging to the Evangelical Lutheran religion. Traditionally, Sweden has been a culturally and racially homogeneous country, as nearly 85% of the population is ethnic Swedes. The economic and social standards of Sweden are among the highest in Europe. The extensive Swedish economic and welfare system is represented by high allocation for health and social issues. This overall context likely provides advantageous conditions for the implementation of large-scale projects such as the Risk Drinking Project that rely on collective responsibility on the part of health care professionals and public support for innovative measures to remedy social ills.

In summary, this article has reviewed key lessons that can be learned from the Swedish Risk Drinking Project and has advanced a number of reasons for its apparent success. However, although the project began in 2004, it is still too early to judge precisely its wider and longer-term effects on hazardous and harmful drinking in Swedish society and too early to tell whether the project can be considered a success in these more demanding terms.
